# Projecting the mental health impacts of the 2025 LA wildfires: lessons from Hurricane Katrina

**DOI:** 10.1088/1748-9326/ae6d16

**Published:** 2026-06-04

**Authors:** Alexis A Merdjanoff, Kate Burrows, Kathleen Lynch

**Affiliations:** 1 School of Global Public Health, New York University, New York, NY 10003, United States of America; 2 Department of Public Health Sciences, University of Chicago, Chicago, IL 60637, United States of America

**Keywords:** mental health, disaster recovery, wildfire

## 1. Introduction

The wildfires that tore through Los Angeles county (LA) in January 2025 are recognized as one of the most destructive wildfire events in U.S. history [1]. The Palisades and Eaton fires burned simultaneously and remained active for over one month, destroying over 16 000 homes, businesses, and schools [1, 2]. Evacuation orders or warnings were issued to over 130 000 people with tens of thousands displaced [3]. Historically, climate-related disasters in the U.S. have rarely caused this level of widespread destruction and mass displacement in densely populated areas like Los Angeles County. Events of this scale are uncommon, meaning there is limited empirical evidence for anticipating long-term mental health needs, including identifying at-risk populations, and determining when and how to implement effective recovery programming. As a result, identifying comparable past events becomes essential.

One such exception is Hurricane Katrina (2005), which displaced nearly half a million residents from their homes in New Orleans and remains the costliest disaster in U.S. history [4]. Katrina can serve as a bellwether for anticipating the long-term mental health needs of LA wildfire survivors, as despite their qualitative differences both disasters produced widespread direct and indirect exposures, including housing damage, displacement, economic loss, strained family dynamics and disrupted social support systems [5–7]. Extensive research on Katrina has demonstrated strong links between these impacts and long-term adverse mental health outcomes, including post-traumatic stress disorder (PTSD), anxiety, depression, and non-specific psychological distress [5, 8]. This longitudinal body of evidence can help guide planning for LA Wildfire survivors by identifying likely mental health outcomes, as well as the types of targeted programming and interventions that may ease long-term impacts.

Although disasters are often treated as isolated cases, the increasing scale and frequency of climate-related disasters demands a more comparative approach. Researchers and policymakers must begin to move beyond viewing each disaster as a unique event and instead prioritize a comparative approach to identify patterns that emerge across different event types, scales and populations. Compiling evidence for drivers of adverse mental health outcomes across hurricanes, wildfires, heatwaves, and other large-scale disasters would allow for more proactive, and ideally more equitable, recovery. The goal of this Perspective is to apply evidence from research on Hurricane Katrina, as it is most similar in scale and scope to the LA wildfires, and identify where and when resources should be used to improve the long-term adverse mental health outcomes of exposure to the LA wildfires.

## 2. Short-term mental health impacts of wildfire exposure

Emerging evidence indicates that exposure to wildfires presents both immediate and long-term risks to mental health. In California, wildfire exposure has been associated with increased prescription of psychotropic medications among impacted residents for up to 6 weeks [9]. Residents who experienced the 2018 Camp Fire—the deadliest fire in California history—had significantly increased risk of anxiety, depression, and PTSD six months following the event [9, 10]. Symptoms related to depression, anxiety, and trauma also have the potential to persist over the long-term, years after the fire is extinguished. A scoping review of wildfire impacts on mental health identified increased rates of PTSD, depression, and generalized anxiety in different populations at follow-up, with direct exposure experiences (e.g. personal witnessing of burning homes, having fear for one’s life or lives of loved ones, losing a loved one, major property damage) significantly linked to PTSD risk years after exposure [11].

Further studies are needed to disentangle the differential psychological impacts of direct and indirect wildfire exposure. Unique from other climate change-amplified disasters, households in the direct path of the fire may face an immediate threat to life, health, and property, while surrounding communities are blanketed in toxic smoke and ash. This presents a challenge to evaluating the extent to which heavy smoke exposure and evacuation may exacerbate poor mental health beyond the trauma stemming from displacement, injury, or direct encounter with flames. Future research is also needed to evaluate the potential neurological and psychological interactions of wildfire smoke. As the frequency and severity of wildfire continues to increase, families and communities will likely experience multiple large-scale, destructive wildfire events in their lifetimes. Thus, it is imperative that public health researchers begin to assess long-term mental health not only of a single event, but also within the context of repeated and cumulative wildfire exposures. Recent research following the camp fires provides context for more immediate mental health needs as communities in Northern California continue to rebuild and recover. In the meantime, researchers must look to prior disasters to hypothesize the outstanding and evolving—and possible long-term—needs to those exposed to the LA wildfires.

## 3. Lessons from Katrina

More than any other disaster in US history, ample longitudinal datasets and multigenerational cohort studies on Hurricane Katrina survivors have facilitated research on long-term effects. Findings show that mental health consequences may continue long after media attention subsides: more than a decade after the storm, there was still an association between exposure to hurricane-related traumatic events and post-traumatic stress symptoms (PTSS) [5]. These findings underscore the importance of long-term care planning, and the rethinking of traditional disaster response timelines following large-scale disasters such as the LA wildfires. Importantly, many PTSD symptoms began more than 6 months *after* Katrina (41.2% in one study), indicating a delayed onset pattern that standard disaster support may fail to address [12]. Particular attention should be paid to those with pre-existing vulnerabilities: for example, pre-Katrina traumatic experiences were associated with long-term post-disaster PD and PTSS [13].

In addition to providing unprecedented knowledge about long-term effects, extensive research on Hurricane Katrina has identified potential mechanisms through which disasters impact mental health. These findings could inform the identification of vulnerable populations in LA who will require additional support, both in the coming months and over the longer term. For example, post-Katrina financial instability was strongly related to PTSD and mental distress, especially for socially vulnerable groups [14]. Research also shows that reduced or delayed access to Federal Emergency Management Agency (FEMA) assistance and the resulting administrative burden on survivors was associated with increased mental distress [15].

Beyond economic instability, a major source of psychological distress resulted from fractured social networks and community dissolution, largely associated with population mobility [16]. Those who returned to New Orleans generally had better mental health outcomes than those who relocated elsewhere or remained unstably housed [8]. Given the magnitude of displacement following the LA fires, these findings are particularly salient, and suggest a need for targeted support among those who relocated, and especially those who do not return. Research on return migration to New Orleans may help identify these individuals: flood exposure was one of the biggest predictors of return migration [7]. Homeownership was also associated with return migration [7], with renters being less likely to move back.

After Katrina, few survivors received the mental health care that they needed, often related to financial and logistical barriers, or perceived need for treatment [17]. Most relied on the general medical sector (rather than specialists) for care, suggesting that integrating mental health resources into primary care settings could be an important way to improve accessibility, prove long-term care options, and reduce stigma associated with treatment [17]. Integrating these services also allows for a more holistic approach to post-disaster recovery by addressing the overlap of physical and mental health. This is particularly relevant given that PTSD symptoms were associated with a higher risk of cardiovascular disease among hypertensive older adults exposed to Katrina [18]. Beyond clinicians’ offices, the Project Fleur-de-Lis program provided school-based mental health care for children affected by Hurricane Katrina, demonstrating the effectiveness of embedding trauma services within community institutions [19]. Mental health care, especially longer-term care, to support those affected by the LA wildfires will likely need to be integrated into existing services to effectively address ongoing psychological needs while overcoming logistical barriers.

Importantly, Hurricane Katrina demonstrated that supporting survivors means not only treating diagnosable conditions, but also addressing psychological wellbeing more broadly, including happiness, contentment, and the capacity to rebuild meaningful lives. Research shows that Katrina survivors reported lower happiness in the year after the storm compared to pre-disaster levels **[**20**]**. Some experienced persistent reductions in happiness for up to four years, while others (especially those with higher pre-disaster happiness and post-disaster social support) recovered more quickly **[**20**]**. Pre- and post-disaster psychosocial resources, including community support, are consistently associated with resilience and recovery **[**21**]**. Identifying both barriers and facilitators of long-term recovery (LTR) specific to wildfire exposure is critical to designing and implementing effective programming.

## 4. Conclusion

Katrina provides a useful foundation for anticipating the long-term mental health challenges likely to emerge following exposure to the 2025 LA wildfires. Despite limited knowledge on the delayed mental health effects of wildfire exposure, Katrina’s scale of destruction and displacement—compounded by population density and limited affordable housing—offers one of the closest parallels available. Insights from this longitudinal body of research highlight three priority areas for further policy implementation and research that address urgent, strategic and long-term needs.

First, evidence from Katrina underscores the need of sustained and long-term mental health funding. Post-disaster mental health services typically expire after only a few months even though impacts can be felt for years [12]. This results in a mismatch of funding timelines versus actual need, as survivors are left without support during the most consequential phases of recovery. These findings suggests that mental health services should be available for at least a year following the LA wildfires, and that funding should be backloaded so that services expand rather than contract as recovery progresses. Integrating mental health services across both disaster response and recovery—via primary care providers, school-based programs, and LTR groups—will help ensure multiple recovery touchpoints that optimize reaching those in need.

Second, funding must support community-based LTR groups. LTR groups serve as critical recovery brokers once immediate response operations finish, functioning as trusted liaisons who help impacted residents navigate insurance claims, state and local recovery programming, and unfamiliar recovery systems. With federal disaster response and recovery assistance facing potential budgetary constraints, including FEMA and community development block grants, the role of LTR groups and Voluntary Organizations Active in Disaster (VOADs), will become more critical. Emerging federal funding barriers means that recovery operations will likely need to be integrated into existing services offered by VOADs and facilitated by LTR groups. Integrating mental health programming into these community-based networks can streamline access to assistance, improve identification of highly impacted residents, and provide opportunities to connect to needed mental health services.

Lastly, there is a pressing need for longitudinal research on the mental health impacts of wildfire exposure to track long-term trajectories. Future studies should prioritize comparability across disaster types by standardizing exposure measures and consistently using the same validated mental health impact measures. This includes a methodological transition from case-based studies to causal inference in the disaster science field, specifically quasi-experimental designs that leverage pre- and post-disaster data over multiple years to provide insight into how disaster exposure can influence mental health **[**22**]**. While prior findings from Katrina allow us to hypothesize probable outcomes from the LA wildfires, wildfire-specific longitudinal data are necessary to clarify both the shared and unique pathways through which mental health impacts unfold. Further, research is needed that focuses specifically on the communities affected by the LA wildfires, as some of the findings from Katrina may be unique to the social and structural context of the Gulf South. Longitudinal evidence would not only help inform the design of future recovery programming but also support the identification of high-risk populations who require ongoing access to mental health services, which is particularly important as wildfire events become more frequent and cumulative in a changing climate.

As one of the most studied disasters in recent history, Hurricane Katrina serves as a foundational case study for understanding the potential long-term mental health impacts of the LA wildfires. However, we acknowledge that the lessons from Katrina may not be comprehensive and that insights may also be drawn from other disasters, particularly in areas such as the role of media on mental health. While direct evidence linking media exposure specifically to Katrina and mental health outcomes is limited, there is evidence that the consumption of media around disasters can impact levels of PTSD and depression **[**23**]**. More generally, media exposure during collective traumas can create a ‘negative cycle’ that can serve as a potential mechanism for prolonged mental health distress **[**24**]**. These findings warrant further exploration in future longitudinal studies, especially concerning social media’s influence on mental health during wildfire events.

While Katrina and the LA wildfires differ by hazard type, geography and population characteristics they share important similarities, including widespread displacement, housing loss, and community disruption, which have been connected to post-disaster mental health trajectories. In the absence of wildfire-specific longitudinal evidence, the lessons from Katrina, gathered from twenty years of research, provide a useful foundation for anticipating possible mental health outcomes and recovery program needs following the LA wildfires.

## Data Availability

No new data were created or analysed in this study.
